# Magnetic Dehydrodipeptide-Based Self-Assembled Hydrogels for Theragnostic Applications

**DOI:** 10.3390/nano9040541

**Published:** 2019-04-03

**Authors:** André Carvalho, Juan Gallo, David M. Pereira, Patrícia Valentão, Paula B. Andrade, Loic Hilliou, Paula M.T. Ferreira, Manuel Bañobre-López, José A. Martins

**Affiliations:** 1 Centre of Chemistry, University of Minho, Campus de Gualtar, 4710-057 Braga, Portugal; andrefcarvalho95@gmail.com; 2 Diagnostic Tools & Methods/Advanced (Magnetic) Theranostic Nanostructures Lab, International Iberian Nanotechnology Laboratory (INL), Av. Mestre José Veiga s/n, 4715-330 Braga, Portugal; juan.gallo@inl.int; 3 REQUIMTE/LAQV, Laboratório de Farmacognosia, Departamento de Química, Faculdade de Farmácia, Universidade do Porto, R. Jorge Viterbo Ferreira, n° 228, 4050-313 Porto, Portugal; dpereira@ff.up.pt (D.M.P.); valentao@ff.up.pt (P.V.); pandrade@ff.up.pt (P.B.A.); 4 Institute for Polymers and Composites/I3N, Department of Polymer Engineering, University of Minho, Campus de Azurém, 4800-058 Guimarães, Portugal; loic@dep.uminho.pt

**Keywords:** naproxen *N*-capped dehydrodipeptides, self-assembled hydrogels, SPION, magnetic hydrogels, MRI, magnetic hyperthermia, drug-delivery

## Abstract

Self-assembled peptide hydrogels have emerged in recent years as the new paradigm in biomaterials research. We have contributed to this field the development of hydrogels based on dehydrodipeptides *N*-capped with naproxen. The dehydrodipeptide hydrogels can be loaded with drugs, thus being potential nanocarriers for drug delivery. In this work novel dehydrodipeptides containing tyrosine and aspartic acid amino acid residues *N*-capped with naproxen and *C*-terminal dehydrophenylalanine were prepared and characterized. Superparamagnetic iron oxide nanoparticles (SPIONs) were incorporated into the dehydrodipeptide-based hydrogels and their effect on the self-assembly, structure and rheological and magnetic properties of the hydrogels was studied. Magnetic hydrogels, with incorporated SPIONs, displayed concentration-dependent *T*_2_-MRI contrast enhancement. Moreover, upon magnetic excitation (alternating magnetic field –AMF–) the SPIONs were able to generate a significant amount of heat. Hence, magnetic hyperthermia can be used as a remote trigger for release of drug cargos and SPIONs incorporated into the self-assembled dehydrodipeptide hydrogels.

## 1. Introduction

Crystalline, single domain iron oxide nanoparticles, usually with core size between 4–18 nm, display superparamagnetism. Superparamagnetic iron oxide nanoparticles (SPIONs) are characterized by fully reversible magnetization curves, i.e., zero coercivity and remanance [1,2] Superparamagnetic properties, allied to amenable methodologies for controlled bottom up synthesis and biocompatibility make SPIONs ideal for biomedical applications—as contrast agents (CA) for magnetic resonance imaging (MRI) and magnetic hyperthermia (MH) [3]. MRI is the leading modality in clinical imaging: anatomical and functional imaging of soft tissues with high spatial-temporal resolution, non-invasive and considered safe owing to use of benign (non-ionizing) radiation, magnetic fields and radiofrequencies. The main limitation of MRI is low detection sensitivity—inherent to the nuclear magnetic resonance (NMR) phenomenon [4]. Paramagnetic metal complexes (Gd^3+^, Mn^2+^, Fe^3+^, organic radicals) and magnetic nanoparticles (i.e., SPIONs) reduce selectively the relaxation times (*T*_1,2_) of the water protons of tissues in their vicinity—Contrast Agents. The efficacy of a CA is measured by the parameter relaxivity (*r*_1,2_)—enhancement of the relaxation rates (*R*_1,2_ = 1/*T*_1,2_) normalized to 1 mM concentration of paramagnetic species. SPIONs are preferentially used as *T*_2_ contrast agents for MRI due to a strong *T*_2_ effect and high ratio *r*_2_/*r*_1_ [5,6]. Size, shape and magnetic composition of the nanocrystal are the main factors that determine the magnetization saturation and the relaxivity of SPIONs [6]. Self-assembly and controlled aggregation of SPIONs is a strategy for development of smart responsive *T*_2_ CA [7]. Upon remote magnetic excitation (AMF) SPIONs generate heat [8,9]. A localized temperature rise above 45 °C induces cell apoptosis [10]. SPIONs is well established in biomedical research and is clinically approved for hyperthermia therapy of cancer [11,12]. Heat generation by SPIONs results from magnetic spin relaxation by Néel and Brownian relaxation mechanisms [13]. The efficacy of magnetic nanoparticles as heat generators is measured by the parameter specific absorption rate (SAR) (named also specific loss power, SLP) [2,9]. The SAR depends on several parameters related to the AMF excitation (frequency and amplitude), magnetic properties of the nanoparticles (density of nanoparticles, saturation magnetization and nanoparticle anisotropy) and environment properties such as medium viscosity and self-assembly of magnetic nanoparticles by dipole-dipole interactions [14,15]. Nanoparticles displaying high SAR can reach the target temperature at lower doses, minimizing potential toxicity to surrounding tissues. Obtaining nanoparticles with high values of SAR requires simultaneous optimization of the parameters that govern SAR [15,16].

Self-assembled peptide hydrogels have emerged in recent decades as the new paradigm in biomaterials research: high water content; *soft materials* with fully organic structure; intrinsic nontoxicity and biocompatibility and fibrillar nanostructure reminiscent of the extracellular matrix [17]. Importantly, the synthesis of hydrogelator peptides (di- and tripeptides) is straightforward and can be automatized by solid-phase synthesis. The properties of the hydrogels can be tuned by design and there is a great variety of possible hydrogelator structures using proteinogenic and non proteinogenic aminoacids [18,19]. Hydrogel assembly/disassembly can be instructed by enzymatic modification of hydrogelator molecules (hydrolysis, phosphorylation/dephosphorylation, etc.) and environmental stimuli (pH, ionic strength, etc.) [20,21]. The self-assembly of hydrogelator peptide molecules is mediated by an ensemble of weak non-covalent intermolecular interactions: hydrogen bonding, *van der Walls* and dispersion interactions, π–π stacking [22]. Our research group has recently described a series of self-assembled hydrogels based on dehydrodipeptides *N*-protected with the Non-Steroidal Anti-inflammatory Drug Naproxen (NSAID). The *C*-terminal dehydroaminoacid residue (dehydrophenylalanine, dehydroaminobutyric acid and dehydroalanine) is prone to make the peptide resistant to proteolysis and restrains the conformational freedom of the peptide [23,24,25]. The naproxen naphtalene bulky aromatic moiety is fundamental for self-assembly as it contributes with intermolecular π–π stacking interactions. The dehydrodipeptide hydrogels revealed an entangled fibrillar structure with nanometric/micrometric dimensions. Interestingly, drug molecules could be incorporated into the dehydrodipeptide-based hydrogels and were found, by FRET experiments, to be in close proximity, presumably associated with the hydrogelator fibers. We have previously proposed that dehydrodipeptide based-hydrogels are potential nanostructured drug delivery agents [24,25].

A remote (external) magnetic field (noninvasive, safe and tunable) is the ideal actuator for in vivo biomedical applications of magnetic materials: as CA for MRI, hyperthermia, drug delivery, etc. Hydrogels can be rendered magneto-responsive by the incorporation of magnetic nanoparticles (γ-Fe_2_O_3_, Fe_3_O_4_, CoFe_2_O_4_, etc.). Magnetic nanoparticles respond to magnetic actuation changing the micro-nanostructure of the hydrogels which in turn leads to modification of the hydrogel properties. Magnetic hydrogels, based on synthetic and natural polymers, have been extensively reported in the literature as potential agents for magnetic field-activated hyperthermia and drug delivery [26,27]. The reversible nature of self-assembly is prone to make supramolecular magnetic hydrogels intrinsically more susceptible to magnetic actuation than polymer-based hydrogels. Bing Xu and co-workers demonstrated that a self-assembled peptide hydrogel, incorporating SPIONs coated with hydrogelator molecules, displays typical magnetorheological behavior, i.e., a gel-sol transition trigged by a non-uniform magnetic field [28]. More recently, Cienfuegos and co-workers reported that the nanostructure and rheological properties of a peptide-based supramolecular hydrogel containing iron nanoparticles can be substantially improved by performing the self-assembly in the presence of a magnetic field [29]. In this work, novel dehydrodipeptide hydrogelators with aspartic acid and tyrosine amino acid residues were synthesized and fully characterized. Dehydrodipeptide hydrogels incorporating SPIONs were prepared and characterized. The effect of SPIONs incorporation on the self-assembly and micro-nanostructure of the hydrogels and on their rheological and magnetic properties, as well as their efficiency as CA for MRI and heat source for magnetic hyperthermia, were studied. Preliminary data on the biological properties of the hydrogelators were collected as well.

## 2. Materials and Methods

*Synthesis*—Hydrogelators **7** and **8** were prepared by synthetic methodologies developed in our laboratory and fully characterized by ^1^H and ^13^C NMR spectroscopy (NMR) and High Resolution Mass Spectrometry (HRM) [23,24,25]. Spectra were acquired on a Bruker Avance III 400 spectrometer, operating at 400.13 MHz and 100.62 MHz, for ^1^H and ^13^C NMR respectively. HRMS data were acquired from the mass spectrometry service of the University of Vigo, Spain. MS was acquired in a Thermo Finnigan LxQ (Linear Ion Trap, San Jose, CA, USA Mass Detector with Electro Spray Ionization (ESI).

*Hydrogel preparation*—Dehydrodipeptides **7** and **8** were weighted into sample vials, water was added and the suspension was adjusted under magnetic stirring to *circa* pH 10 (pH meter) by addition of NaOH (1M). The resulting solutions were sonicated for 30 s and d-glucono-δ-lactone (GDL) was added under magnetic stirring (around 10 s). The solutions were left standing overnight at room temperature (20–25 °C). Hydrogels loaded with SPIONs were prepared following the same methodology. A SPIONs solution was added to the hydrogelator suspension in water, followed by pH adjustment to *circa* 10, sonication and the addition of GDL [25].

*Rheology*—Hydrogel formation kinetics and mechanical spectra were measured with a stress-controlled rotational rheometer (MCR300, Anton Paar, Graz, Austria). Liquid samples, prepared as described above, were loaded into the Couette geometry of the rheometer. The temperature was kept at 25 °C during testing. For measuring gel kinetics, a small amplitude oscillatory shear strain, with a frequency of 1 Hz and an amplitude varying from 0.0001% to 1%, depending on the SPIONs loading in samples, was applied for 60,000 s. After the kinetic experiments, mechanical spectra were recorded using the same strain amplitude as in the kinetics tests, and ramping the frequency from 100 Hz down to 0.01 Hz. Finally, a sweep in the strain amplitude was performed from 0.001% to 100% and both storage (G′) and loss (G″) shear moduli were measured.

*Transmission Electron Microscopy*—TEM images were obtained on a JEOL (Tokyo, Japan) JEM-2100 microscope at an accelerating voltage of 200 kV. Samples for TEM were prepared as described for circular dichroism (CD) spectroscopy. A small volume of sample (10 µL) was placed on the top of a carbon-coated grid and left for 30 s. Excess water was removed using filter paper. UranyLess stain (10 µL) was added to the grid and left for 30 s. The grid was washed twice with water.

*CD spectroscopy*—CD spectra were recorded under N_2_ on a Jasco (Tokyo, Japan) J815 CD spectrometer using solutions of hydrogelators **7** and **8** (0.02 wt%) without and with 10% SPIONs.

*Magnetization studies*—Magnetization curves were recorded at 5 and 260 K as function of the applied magnetic field (up to ±2 T). Zero-field-cooled (ZFC, H = 0) and field-cooled (FC, H = 100 Oe) magnetization curves were recorded over the temperature range of 5–300 K. A superconducting quantum interference device magnetometer (SQUID-VSM, Quantum Design, San Diego, CA, USA) was used for all experiments.

*Magnetic Resonance Imaging*—MRI was performed in a 3.0 T horizontal bore MR Solutions (Guildford, UK) Benchtop MRI system equipped with 48 G/cm actively shielded gradients. To image the samples, a 56 mm diameter quadrature birdcage coil was used in transmit/receive mode. Samples for phantom measurements, SPIONs in water and SPIONs incorporated into hydrogels **7** and **8** at different concentrations (0–0.43 mM Fe), were prepared with a total volume of 200 µL in 300 µL tubes. All MR images of the phantoms were acquired with an image matrix 256 × 252, field of view (FOV) 60 × 60 mm, 3 slices with a slice thickness of 1 mm and 1 mm slice gap. For *T*_2_-weighted imaging, fast spin echo (FSE) sequences with the following parameters were used: TE = 15 ms, TR = 1500 ms, NA = 12, AT = 24 m 48 s. For *T*_1_-weighted imaging a FSE sequence with TE = 11 ms, TR = 400 ms, NA = 12 and AT = 7 m 34 s was used.

*Hyperthernia studies*—SPIONs in water and SPIONs (25–35%) incorporated into hydrogels **7** and **8** were studied as magnetic hyperthermia effectors using a nB nanoScale Biomagnetics (Zaragoza, Spain) DM-100 instrument.

*Biological studies*—The toxicity of hydrogelator molecules **7** and **8** was evaluated in vitro towards macrophage cells (RAW 264.7 cell line) and towards the human non-cancer fibroblast cell line MRC-5 using the MTT reduction assay. The effect of hydrogelators **7** and **8** on the NO levels on RAW 264.7 cells challenged with LPS [30] and the ability of these molecules to inhibit key enzymes in the inflammatory cascade, namely 5-lipooxygenase, which is involved in the synthesis of leukotrienes, and cyclooxygenase-2, which is involved in the synthesis of prostaglandins, was evaluated as well. The LOX inhibition assay [31] was performed with soybean lipoxygenase. The COX-1/COX-2 inhibition assay was performed using a commercial screening assay kit (Cayman chemical, Ann Arbor, MI, USA).

## 3. Results and Discussion

### 3.1. Synthesis

**Scheme 1:**
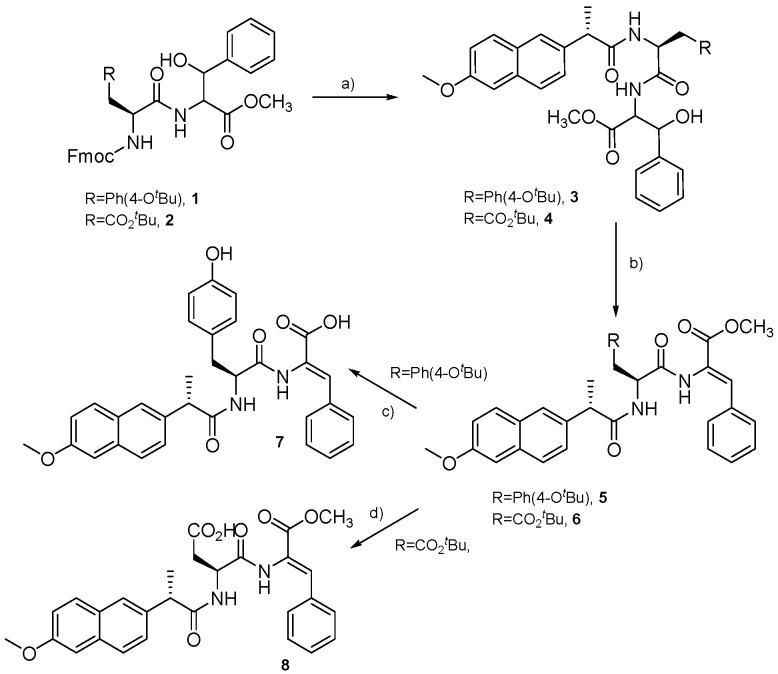
Synthesis of dehydrodipeptides *N*-conjugated with naproxen **7** and **8**. a) i. piperidine in DMF, ii. naproxen chloride, NEt_3_; b) i. Boc_2_O/DMAP, ii. TMG (2%); c) i. TFA, ii. NaOH d) TFA.

Dehydrodipeptides naproxen tyrosyldehydrophenylalanine [Npx-l-Tyr-*Z*-ΔPhe-OH (**7**)] and aspartyldehydrophenylalanine [Npx-l-Asp-*Z*-ΔPhe-OH (**8**)] *N*-protected with the non-steroidal anti-inflammatory drug (NSAID) naproxen (Npx) were prepared using a solution strategy developed by the research group (Scheme 1) [23,24,25].

Dehydrodipeptides **7** and **8** were obtained in moderate yield and were fully characterized by ^1^H and ^13^C bi-dimensional NMR techniques. The Z stereochemistry of the dehydrophenylalanine residue was established by NOE experiments. The dehydrophenylalanine residue (ΔPhe) is prone to make the peptide recalcitrant to proteolysis and confine the available conformational space [23,32]. The synthetic pathway developed for dehydrodipeptide Npx-l-Asp-*Z*-ΔPhe-OH (**8**) allows either orthogonal deprotection of the *C*-terminal methyl ester or deprotection of the aspartyl side-chain β-*tert*-Butyl ester. Orthogonal deprotection of the aspartyl side-chain β-*tert*-Butyl ester was deemed sufficient to render the peptide water soluble for self-assembly studies.

### 3.2. Preparation of Hydrogels

A fine balance between hydrophilicity (and water solubility) and hydrophobicity is critical for peptide self-assembly [22]. Dehydrodipeptides **7** and **8** showed limited solubility in buffer solutions in the physiological pH range (6–8), but could be rendered soluble upon the adjustment of water dispersions (0.4–0.8 wt%; 4–8 mg/mL) to pH 10 with diluted NaOH (1 M). Gelation of dehydrodipeptides **7** and **8** was trigged by slow pH dropping (to pH 6–7) induced by hydrolysis of added GDL (0.3 wt%; 3 mg/mL).

The minimal concentration required for peptide gelation, critical gelation concentration (cgc), was determined for dehydrodipeptides **7** and **8** as 0.4 wt% (4 mg/mL) (Figure S1). This value is of the same order of magnitude as that reported previously for other dehydrodipeptides [23,24,25]. We envisaged that incorporation of SPIONs into hydrogels **7** and **8** could endow them with magnetic properties. Water stabilized SPIONs with a polyacrylic acid corona (core size 8 nm; hydrodynamic diameter *circa* 100 nm) were used to minimize the likelihood of nanoparticle aggregation and precipitation (Figure S2 and Table SI) [1,2]. Hydrogels **7** and **8** (0.8 wt%) loaded with SPIONs (25–35% m/m; in relation to the hydrogelator weight) were prepared by the GDL method. A solution of SPIONs was added to suspensions of hydrogelators **7** and **8** in water and adjusted to pH 10, followed by addition of GDL. Hydrogels, homogeneous to visual inspection, were obtained in all cases, indicating that nanoparticle precipitation did not occur during gelation. Hydrogels were stable for extended time periods (Figure S3). The incorporation of SPIONs into the hydrogels resulted in slower gelation kinetics and weaker (less elastic) gels. Hydrogels **7** and **8**, with SPIONs loadings in the range 25%–35%, could not be obtained at hydrogelator concentrations 0.4 wt% (cgc). A 0.8 wt% hydrogelator concentration was required for obtaining hydrogels **7** and **8** with 25–35% SPIONs. As the hyperthermia studies (see section on magnetic hyperthermia) required SPIONs in a concentration range of 25%–35%, all studies were carried out with both hydrogels at 0.8 wt% hydrogelator concentration.

### 3.3. TEM Studies 

The micro-nanostructure structure of hydrogels **7** and **8** was studied by transmission electron microscopy (TEM). Images were obtained with diluted solutions of hydrogelators **7** and **8** (0.02 wt%) prepared under the same set of conditions as those used for hydrogel preparation. Self-assembly of dehydrodipeptides **7** and **8** results in entangled fibril structures (Figure S4). Gelation is the result of water trapping by the fibril network. The fibers formed by self-assembly of dehydrodipeptide **7** display a thickness around 10–20 nm and its length varies from approximately 0.2 to 0.4 μm. Dehydrodipeptide **8** shows fibers with diameters around 10–20 nm and lengths between 70 nm and 0.8 μm. TEM images of hydrogels **7** and **8** with incorporated SPIONs could not be obtained.

### 3.4. Circular Dichroism

**Figure 1:**
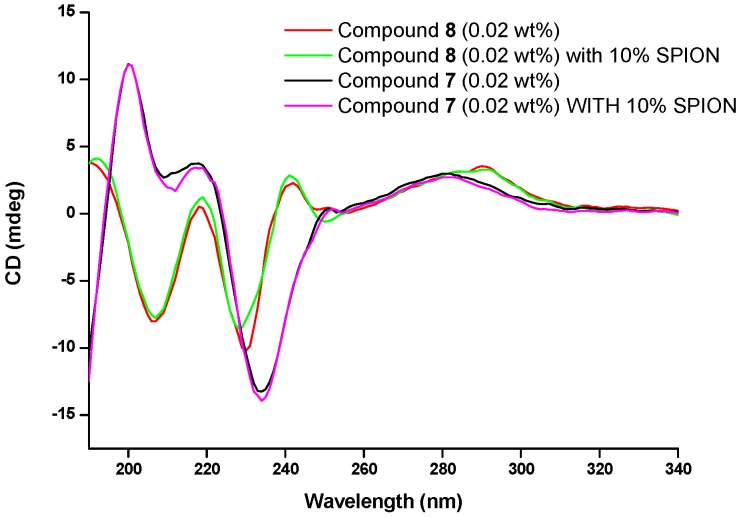
CD spectra of solutions of dehydrodipeptides **7** and **8** (0.02 wt%) without and with 10% SPIONs.

CD spectroscopy is a valuable tool for evaluating the secondary structure, folding and binding properties of peptides and proteins [33]. The possibility of detecting chiral self-assembly of molecular units is especially relevant for the characterization of self-assembled peptide hydrogels [34]. CD spectra were acquired with solutions of hydrogelators **7** and **8** (0.02 wt%), without and with 10% SPIONs (Figure 1).

The CD spectrum of dehydrodipeptide **7** exhibits bands at around 200 and 220 nm (positive bands) and at 230 nm (broad negative band). The CD spectrum of dehydrodipeptide **8** shows a positive band below 200 nm and two negative bands at 210 nm and 230 nm. The positive broad band at around 280 nm, present in the CD spectra of both hydrogels, can be attributed to chiral self-assembly of the naphthalene moieties of the Npx group [23,24,25]. Addition of SPIONs results in broadening of the CD spectra of both hydrogels, although the general spectral features seem unchanged. Hence, incorporation of SPIONs seems not to change the structure of the self-assembled peptide nanofibers [29].

### 3.5. Rheology

**Figure 2:**
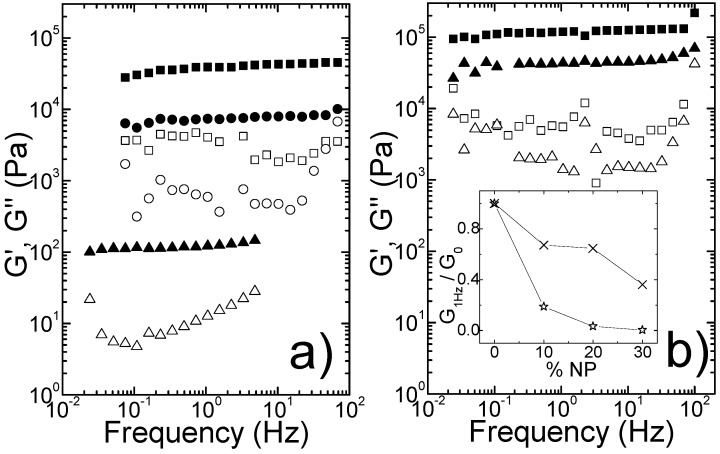
Mechanical spectra (solid symbols: G′; empty symbols: G″) of hydrogel **7** (**a**) and hydrogel **8** (**b**) containing 0% NP (squares), 10% NP (circles) and 30% NP (triangles). Inset: effect of SPIONs content (in %) on the shear storage moduli of hydrogels **7** (stars) and hydrogels **8** (crosses) measured at 1 Hz, G_1Hz_, and rationalized by the corresponding value of neat gels, G_0_.

The structural buildup of hydrogel **7** (0.8 wt%) without SPIONs and with SPIONs (20–35%) was followed by rotational rheometry. Incorporation of SPIONs into hydrogel **7** leads to longer gelation half times, i.e., the time for attaining half of the equilibrium value for the gel elastic modulus G′ (Figure S5). As a general trend, the gelation half time increases with the SPIONs content in the hydrogel (inset to Figure S5). The mechanical spectra of equilibrated hydrogels **7** and **8** formulated without and with SPIONs are presented in Figure 2. It is noteworthy that the rheological properties of hydrogels **7** and **8** are not affected by the temperature up to 40 °C (see Figure S6).

For hydrogel **7** formulated without SPIONs and all hydrogels **8** a waiting time of 6.104 s with no mechanical excitation was applied before performing the frequency sweep, in order to avoid any strain-induced structure and gel elasticity.

Hydrogels **8** was revealed to be significantly more elastic (stronger gels) than hydrogels **7** (Table 1).

The minimum in the frequency dependence of the shear loss modulus G″ is shifted to lower frequencies upon the addition of SPIONs, implying that the nanoparticles modulates the gel structure of both hydrogels. Hydrogels **7** were revealed to be more sensitive to the addition of SPIONs. This result is in accordance with the SPIONs-induced delayed structural buildup (Figure S5). Most importantly, the addition of SPIONs weakens gel elasticity-the shear storage modulus measured at 1 Hz (G_1Hz_) decreases with the increasing SPIONs content. The drop in gel elasticity upon the addition of SPIONs is more significant for hydrogels **7** comparing to hydrogels **8** (inset to Figure 2).

The strain dependence of the storage and loss moduli for hydrogels **8** (similar results were obtained for hydrogels **7**) shows that at low strains both G′ and G″ moduli are constant, as expected for the linear regime of viscoelasticity. G″ goes through a maximum at large strain before dropping and crossing over with the storage modulus G′ (Figure S7). The local maximum in G″ in the strain regime, as G′ increases with increasing strain, reveals a hardening behavior that is the hallmark of many biological networks made by the assembly of fiber-like objects (inset to Figure S7) [35,36]. Strain hardening with concentration is also found in colloidal gels [37], gelatin gels [38] and some polysaccharide gels [39]. Considering a fiber-like structure of self-assembling units, which rule the elasticity of the gels over SPION-SPION contacts, the picture emerging from the rheological data is that the presence of SPIONs swells the mesh size of the structure and slows down the formation of elastically effective fiber-fiber contacts. Alternatively, the decrease of the gel elasticity with increasing SPIONs content could result from a stiffening effect of the SPIONs on the fibers without changing the mesh size [36].

### 3.6. Magnetic Properties

**Figure 3:**
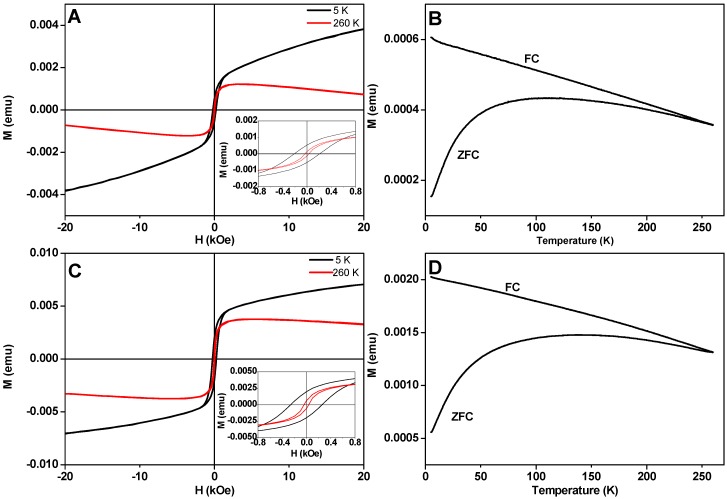
Hysteresis loop at 5 and 260 K for hydrogel **7** (**A**) and hydrogel **8** (**C**) with 10% of incorporated SPIONs. ZFC-FC (100 Oe) magnetization curves for hydrogel **7** (**B**) and **8** (**D**) with 10% incorporated SPIONs. Insets: High magnification at low magnetic fields.

The magnetic properties of both non-magnetic (without SPIONs) and magnetic (with SPIONs) hydrogels **7** and **8** (0.8 wt%) were evaluated by magnetic measurements in a superconducting quantum interference device (SQUID-VSM) magnetometer as a function of the temperature (5–260 K) and applied magnetic field (up to ±2 T) at 5 and 260 K. The hysteresis loop for hydrogel **8** (0.8 wt%) without SPIONs at 260 K confirms the expected diamagnetic behavior of the hydrogels. The paramagnetic behavior observed at 5 K was confirmed as arising from the sample holder (Figure S8). Hydrogels **7** and **8** (0.8 wt%) loaded with 10% SPIONs exhibit superparamagnetic behavior at 260 K indicated by negligible hysteresis and remnant magnetization at zero field of the hysteresis loop (Figure 3).

Superparamagnetism is typical of single domain magnetic nanoparticles, i.e., with diameter bellow the single-to-multi domain critical size (around 20–25 nm for magnetite) [40,41]. The decrease of the magnetization observed at high magnetic fields still shows a significant diamagnetic contribution (coming from the organic matrix) to the overall paramagnetism. At 5 K, a ferromagnetic-like contribution (coercive field, Hc = 210 Oe) is indicated by an open hysteresis loop due to the presence of a magnetically blocked state. The linear increase of the magnetization at high applied magnetic fields results from a dominant paramagnetic contribution. The ZFC-FC (H = 100 Oe) magnetization curves confirm the magnetic behavior observed in the hysteresis loops. The ZFC and FC (100 Oe) curves for hydrogel **8** without SPIONs follow a paramagnetic (Curie law) decay with temperature (Figure S8). The ZFC and FC curves for hydrogels **7** and **8** loaded with 10% SPIONs, indicate a magnetic transition from a magnetic blocked state (ferromagnetic-like behavior) to a superparamagnetic state above the blocking temperature (T_B_) (T_B_ = 138 and 136 K for hydrogels **7** and **8** with 10% SPIONs, respectively). The blocking temperature, defined as the maximum of the ZFC curve, indicates the temperature at which the thermal energy becomes comparable to the anisotropy energy barrier [42]. Interestingly, the divergence between the ZFC and the FC curves in the whole range of temperature, up to 300 K, is also indicative of strong magnetic dipolar interactions between the SPIONs in the gel network. In addition to the presence of a distribution of magnetic anisotropies in the sample, likely due to particle size polydispersity, large differences between T_B_ and ZFC-FC irreversibility temperature (T_irr_) indicate strong magnetic coupling between nanoparticles. In a closer inspection, we can observe that the magnetization monotonically increases with the temperature down to 2 K, further pointing to a strong interacting magnetite nanoparticle system and highlighting the presence of significant dipolar magnetic interactions. This magnetically strong interacting-like behavior differs from the collective behavior observed in other magnetic nanoparticles-based systems, such as magnetite nanoparticles arrays and magnetic zeolites, where both ZFC and FC magnetization curves decrease below T_B_ [43,44]. The rheology experiments support the fibril nature of the hydrogels established by TEM and indicate that the SPIONs incorporated into the hydrogels are likely distributed into the *water pools* created by fiber entanglement. The alternative rheological view of the hydrogels, that the nanoparticles decorate the hydrogel fibers, seems unlikely given the expanded anionic polyacrylic acid corona surrounding the SPIONs. The magnetic results seem to support the rheological picture, pointing also to random distribution of the SPIONs inside the hydrogel matrix which allows the nanoparticles to interact strongly, rather than this magnetic coupling being induced by the fibrillar nano-structure of the hydrogels.

### 3.7. Magnetic Resonance Imaging

**Figure 4:**
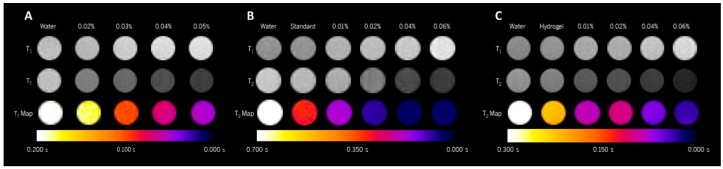
*T*_1w_- and *T*_2w_-MRI phantom images and *T*_2_ relaxation maps (25 °C, 3 T): (**A**) SPIONs in water (**B**) SPIONs incorporated into hydrogel **7** (0.8 wt%); (**C**) SPIONs incorporated into hydrogel **8** (0.8 wt%). The *T*_2_-MRI relaxation map was acquired using the MEMS (multi-echo-multi-spin) sequence.

SPIONs selectively reduce the transverse relaxation times (*T*_2_) of the water protons resulting in dark images in MRI under *T*_2_-weighted (T_2w_) sequences, thus being considered *T*_2_ contrast agents [6,45]. However, it has been already observed that metal doping and particle size effects (surface/volume ratio) can originate predominant *T*_1_ or dual *T*_1_/*T*_2_ behavior in iron oxide based nanoparticles [46,47]. The performance of the SPIONs as contrast agents for MRI, in both aqueous solution and incorporated into hydrogels **7** and **8**, was evaluated by *T*_1w_- and *T*_2w_-MRI images and *T*_2_ relaxation maps (25 °C, 3 T) (Figure 4).

Figure 4A clearly shows a concentration-dependent dark effect under a *T*_2_-MRI sequence for aqueous dispersions of SPIONs. The MRI contrast under *T*_1w_ sequence showed no significant differences between the different nanoparticle concentrations in relation to the water control. This confirms the SPIONs used in this study as being *T*_2_-MRI enhancers. The efficacy of the SPIONs as transverse paramagnetic water relaxers was evaluated by the parameter transverse relaxivity, *r*_2_ (Table 2; Figure S9), calculated from the *T*_2_-MRI maps at 3 T and room temperature.

The SPIONs in aqueous solution displays a considerable transverse relaxivity (*r*_2_ = 122 mM^−1^s^−1^ at 3 T) comparable to that exhibited by other materials with similar core size and magnetic composition (e.g., Feridex, *r*_2_ = 120 mM^−1^s^−1^, 1.5 T) [48]. The relaxivity of the SPIONs used in this work is likely still limited by the small size of the magnetic core (~8 nm) and inherent surface *spin canting* effects [49].

Incorporation of the SPIONs into hydrogels **7** and **8** results in up to a 4-fold reduction of *r*_2_ (*r*_2_ = 30 mM^−1^s^−1^, 3 T, for hydrogel **7**; *r*_2_ = 44 mM^−1^s^−1^, 3 T, for hydrogel **8**) (Table 2). The enhancement of the relaxation rates of the water protons brought about by SPIONs results from the relative translational diffusion of the water molecules and magnetic nanoparticles, reminiscent of the outer-sphere Solomon-Bloembergen-Morgan relaxation mechanism for paramagnetic metal complexes [4,6]. Hence, the transverse relaxivity *r*_2_ is compromised by all effects that restrict water diffusion. The TEM images show that the self-assembled hydrogels **7** and **8** are made of a network of entangled nanosized fibers. The rheology results suggest that the SPIONs incorporated into the hydrogels are likely distributed into the *water pools* created by fiber entanglement. This is further supported by the strong magnetic dipolar particle interaction deduced from the magnetic properties, as the magnetic particles would be confined in the aqueous regions delimited by the peptide fibers. Restricted water diffusion inside the hydrogel water compartments is very likely the cause of the observed low *r*_2_ relaxivities. Aggregation of the SPIONs in the aqueous hydrogel compartments is unlikely, owing to steric and electrostatic stabilization resulting from the nanoparticles bulky PAA anionic corona (Table SI) [2]. This dramatic reduction of *r*_2_ brings an increase of the *r*_2_/*r*_1_ ratio, which interestingly enables dual *T*_1_/*T*_2_-MRI performance. Dual CAs are gaining increasing attention, as they can be separately used under both *T*_1w_ and *T*_2w_ imaging sequences and so contribute to identify interferences and approach a more accurate diagnosis of disease. In addition to the observed darker contrast with increasing SPIONs concentration under a T2w sequence, phantom images of hydrogels **7** and **8** (Figure 4B,C, respectively) show a concentration-dependent positive contrast when acquired with a T1w sequence, which significantly becomes brighter as the particle concentration increases compared to the water control. Disassembly of magnetic hydrogels **7** and **8** implanted in vivo could be monitored overtime. Hydrogel disassembly trigged by environmental/biochemical cues or remote triggers (magnetic hyperthermia) could also report on the drug release and on the hydrogel environment.

### 3.8. Hyperthermia Studies

**Figure 5:**
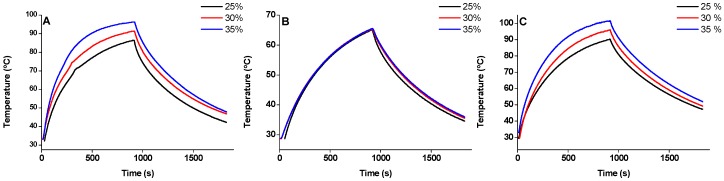
Magnetic hyperthermia curves for SPIONs (25–35%) in water (**A**) and SPIONs (25–35%) incorporated into hydrogels **7** (**B**) and **8** (**C**) (0.8 wt%) upon magnetic actuation (H = 240 G, *f* = 869 kHz).

The concentration of SPIONs deployed for the MRI studies (0.01–0.06%) revealed unsuitable (too low) for hyperthermia purposes. Hydrogels **7** and **8** were studied at 0.8 wt% concentration to ensure gelation with SPIONs in the concentration range 25–35%, as previously explained. SPION concentrations in the range 25–35% were required for the hydrogels undergo gel-solution phase transition upon magnetic actuation (H = 240 G, *f* = 869 kHz). The capacity of the SPIONs in water and incorporated into hydrogels **7** and **8** (0.8 wt%) to transduce magnetic power (H = 240 G, *f* = 869 kHz) into heat was evaluated by the SAR parameter (Figure 5, Table 3 and Table SII).

The specific absorption rate SAR (W/g) parameter was calculated by the initial slope method from the magnetic hyperthermia curves (Table 3, Table SII) [14].

The SPIONs in water display a SAR value (300 W/g; H = 240 G, *f* = 869 kHz), within the range expected for Fe_3_O_4_ nanoparticles with a core size around 8 nm [50]. Whereas incorporation of SPIONs into hydrogels **7** leads to a very substantial reduction (over 50%) of their efficacy (SAR) as heat generators, hydrogel **8** has no effect on the SAR. The heating properties of SPIONs activated by an oscillating magnetic field result from Néel and Brownian relaxation of the magnetic moment of the nanoparticles [8,9]. The significant decrease of SAR for the SPIONs incorporated into the hydrogels **7** is likely to result from a reduction of the contribution of the Brownian relaxation mechanism to the overall relaxation. Néel relaxation, less effective than Brown relaxation for small magnetite nanoparticles, presumably becomes the dominating heat generating mechanism when the SPIONs are incorporated into hydrogel [8]. This suggests that the SPIONs incorporated into hydrogels **7** have less mobility than when incorporated into hydrogels **8**. SPIONs incorporated into hydrogels **8** seem to be in a *quasi* aqueous environment. The rheological experiments showed that the elasticity of both hydrogels **7** and **8** is reduced by incorporation of SPIONs, suggesting that the SPIONs swell the mesh size of the hydrogel fibril network. As this effect is more pronounced for hydrogel **7** the hydrogel *water pools* are likely to be bigger for hydrogel **8**. This result is in accordance also with the higher relaxivity measured for SPIONs incorporated in hydrogel **8**. Using hydrogelator concentrations 0.8 wt% and a SPIONs content of 25%–35% magnetic excitation (H = 240 G; *f* = 869 kHz) is still efficient in inducing a phase transition that leads to hydrogel collapse. Hence, the hydrogels loaded with SPIONs can be potential drug carriers for magnetically-triggered drug delivery.

### 3.9. Biological Studies

**Figure 6:**
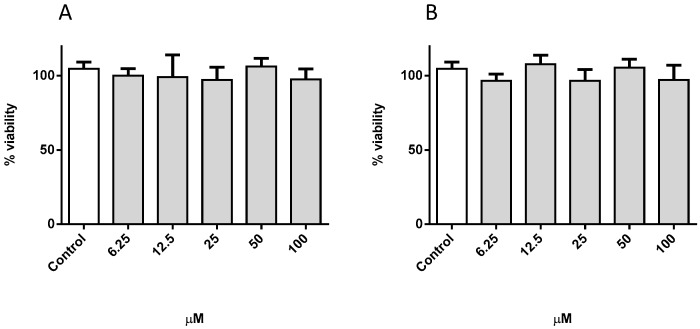
Viability of RAW 264.7 cells incubated with hydrogelators **7** (**A**) and **8** (**B**). Data represent the mean ± standard deviation of the mean of three independent experiments, performed in triplicate.

The NSAID properties of naproxen are well established in the clinical practice. Naproxen is a potent non-selective inhibitor of both COX-1 and COX-2 enzymes [51]. Hydrogelator molecules **7** and **8** are unique molecular entities, but can also be considered naproxen conjugates with a dehydrodipeptide [52]. In this work we evaluated the toxicity of hydrogelator molecules **7** and **8** in vitro towards macrophage cells (RAW 264.7 cell line) and also their potential anti-inflammatory properties. As shown in Figure 6, both molecules are non-toxic in concentrations up to 100 µM. In addition to macrophages, we also evaluated the toxicity of the hydrogelators towards the human non-cancer fibroblast cell line MRC-5 (Figure S10). No toxicity has been observed in this concentration range.

As experimental model for the evaluation of anti-inflammatory activity, we have used RAW 264.7 cells challenged with the pro-inflammatory molecule lipopolysaccharide (LPS), a molecule derived from Gram negative bacteria, which elicits a number of genetic, biochemical and morphological changes in macrophages that result in a pro-inflammatory phenotype. Among the pro-inflammatory mediators that are upregulated, nitric oxide (NO) is the most widely used for screening proposes.

**Figure 7:**
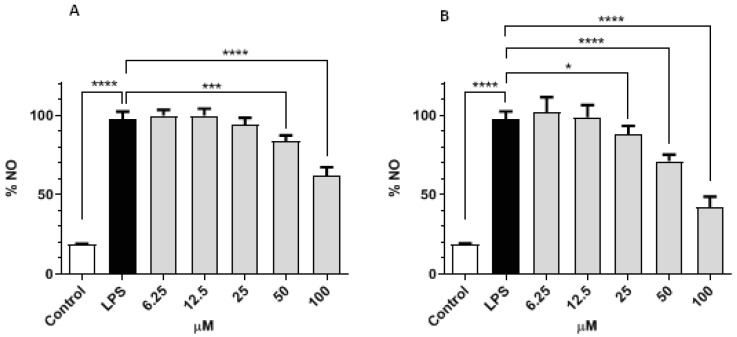
Effect of compounds **7** (**A**) and **8** (**B**) in the NO levels of LPS-challenged RAW 264.7 cells. Data represent the mean ± standard deviation of the mean of three independent experiments, performed in triplicate. * *p* < 0.05, *** *p* < 0.001, **** *p* < 0.0001.

Incubation of LPS-challenged RAW 264.7 cells with compounds **7** and **8** significantly decreased NO levels when compared with non-treated cells (Figure 7). While both molecules displayed a concentration-dependent effect, compound **8** displayed higher potency, as it showed capacity to lower NO levels in cells in concentrations as low as 25 µM and resulted in over a 50% reduction at the highest concentration tested.

**Figure 8:**
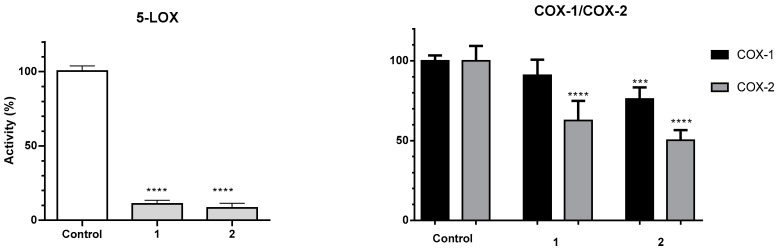
Inhibitory effects of **7** and **8** towards 5-LOX activity. Data represents the mean ± standard deviation of the mean of three independent experiments, performed in triplicate. *** *p* < 0.001, **** *p* < 0.0001.

In addition to the effect found towards NO levels, we were interested in evaluating the ability of these molecules to inhibit key enzymes in the inflammatory cascade, namely 5-lipooxygenase, which is involved in the synthesis of leukotrienes, and cyclooxygenase-2, which is involved in the synthesis of prostaglandins. In fact, dual inhibition COX/LOX has been proposed as a smart strategy to optimize the anti-inflammatory activity of NSAID inhibitors [53]. At the highest concentration that was shown to be devoid of toxicity, 100 µM, both hydrogelators **7** and **8** almost completely inhibited the activity of LOX (Figure 8).

The inhibitory activity of compounds **7** and **8** towards COX-1 and COX-2 enzymes was shown to be moderate at the concentrations studied. It is interesting to note that compounds **7** and **8** show some degree of selectivity towards the enzyme COX-2, while naproxen is non-selective [19]. This is particularly important if we consider that inhibition of COX-1 is associated with many of the side-effects of NSAIDs [54].

Compounds **7** and **8** show interesting anti-inflammatory properties, demonstrating that nontoxic hydrogelator molecules can combine a structural function with intrinsic pharmacological activity.

## 4. Conclusions

In this work we demonstrate that SPIONs incorporated into dehydrodipeptide-based self-assembled hydrogels retain their magnetic properties despite the strong diamagnetic contribution from the organic matrix of the hydrogel. Magnetic hydrogels with a significant, SPIONs concentration-dependent, dual *T*_1_/*T*_2_ MRI contrast were obtained, suggesting that the fate hydrogels **7** and **8** implanted in vivo could be monitored by MRI. Moreover, upon magnetic excitation with an AMF the SPIONs generated a significant amount of heat, sufficient to induce in vitro a gel to solution phase transition of hydrogels **7** and **8**. Thus, we propose that MH could be used as a remote trigger for release of SPIONs and drug cargos incorporated into dehydrodipeptide hydrogels. Nontoxic hydrogelator molecules can combine a structural function with intrinsic pharmacological activity allowing combined hyperthermia with multi-drug therapy and imaging. The AMF-triggered release of SPIONs from the magnetic hydrogel could be used as a self-reporting mechanism (darker MRI contrast as consequence of the recovery of the original SPIONs transverse relaxivity, *r*_2_), constituting as a step forward towards image-guided drug delivery and the design of more efficient theragnostic platforms.

## Figures and Tables

**Table 1:** Effect of SPION content in G′ and G″ for hydrogels **7** and **8**.

	Hydrogel 7		Hydrogel 8	
SPIONs %	G′ (Pa)	G″ (Pa)	G′ (Pa)	G″ (Pa)
0	39,300	3530	119,000	5520
10	7390	593	80,000	3400
20	1310	30	76,600	3300
30	122	13	43,500	1400

**Table 2:** SPIONs transverse relaxivity (25 °C, 3 T) in water and following incorporation into hydrogel **7** and hydrogel **8** (0.8 wt%).

	SPIONs in Water	SPIONs Incorporated into Hydrogel 7	SPIONs Incorporated into Hydrogel 8
***r*_2_ (mM^−1^ s^−1^) (25 °C; 3 T)**	122	30	44

**Table 3:** SAR for SPIONS in water and hydrogel **7** and **8** (0.8 wt%) loaded with SPIONs (25–35% m/m).

SPIONs (%)	SAR (w/g)		
	SPIONs in Water	SPIONs in Hydr. 7	SPIONs in Hydr. 8
25	357	161	347
30	325	100	305
35	227	65	240

## Supplementary Materials

The Supplementary Materials are available online at https://www.mdpi.com/2079-4991/9/4/541/s1.
