# Supplementary material for: Magnetic Dehydrodipeptide-Based Self-Assembled Hydrogels for Theragnostic Applications
Source: Nanomaterials (Basel). 2019 Apr 3;9(4):541. doi: 10.3390/nano9040541 (PMC6523327; doi:10.3390/nano9040541)
Supplement: Supplementary file 1 [file nanomaterials-09-00541-s001.pdf]

Supportting Information

Magnetic dehydrodipeptide-based self-assembled hydrogels for theragnostic applications

Synthesis

^{1}H and ^{13}C NMR spectra were recorded on a Bruker Avance III at 400 and 100.6 MHz, respectively. ^{1}H–^{1}H spin–spin decoupling and DEPT θ 45° were used. HMQC and HMBC were used to attribute some signals. Chemical shifts (δ) are given in parts per million (ppm) and coupling constants (*J*) in Hertz (Hz). High resolution mass spectrometry (HRMS) data were recorded by the mass spectrometry service of the University of Vigo, Spain. Column chromatography was performed on Macherey–Nagel silica gel 230–400 mesh. Petroleum ether refers to the boiling range 40–60 °C.

*Synthetic procedures for preparation of hydrogelators **7** and **8***

Synthesis of Npx-L-Tyr(O^{t}Bu)-Phe(β-OH)-OMe, 3: Fmoc-Tyr(O^{t}Bu)-Phe(β-OH)-OMe, **1** (1 mmol, 0.64 g) was dissolved in DMF (2 mL) and piperidine (0.5 mL) was added. The mixture was left stirring for 10 minutes until a white precipitate started to form. Ethyl acetate (50 mL) was added and reaction mixture was washed with distilled water (3 x 50 mL). The organic phase was dried and the solvent removed to give *N*-deprotected dipeptide as an oil (1.27 g). This compound was used without purification. Thus, the oil was dissolved in dry DCM (7 mL) and (S)-(+)-naproxen chloride (1 mmol , 0.25g) and NEt_3 (2.1 eq., 2.1 mmol, 0.3 mL) were added. The reaction mixture was left stirring overnight (18 h). Removal of the solvent at reduced pressure gave a residue that was partitioned between ethyl acetate (50 mL) and $KHSO_4$ (30 mL, 1 M). The organic phase was thoroughly washed with $KHSO_4$ (1 M), $NaHCO_3$ (1 M), and brine (3 × 30 mL, each) and dried with $MgSO_4$. Removal of the solvent afforded compound **3** as diastereomeric mixture (0.56 g, 89%). ^{1}H NMR (400 MHz, DMSO ,δ):1.30 and 1.31 (9H, 2s, $C(CH_3)$, 1.60 and 1.57 (3H, 2d, *J* = 7.2 Hz, CH_3 Npx), 2.71-2.88 (2H, m, β-CH Tyr), 3.61 and 3.68 (3H, 2s, OMe CO_2Me), 3.82-3.93 (4H, m, CH Npx + OCH_3 Npx), 4.55-4.61 [2H, m, α-CH Phe(β-OH)] + α-CH Tyr), 5.10-5.17 [1H, m, Phe(β-OH)], 6.82 (2H,d, *J* = 8.4, ArH Tyr), 7.01 (2H,d ,*J* = 8.4 Hz, ArH Tyr) 7.12-7.76 [13 H, m, ArH + NH Tyr + Phe(β-OH)]

Synthesis of Npx-L-Tyr(O^{t}Bu)-*Z*-ΔPhe-OMe, 5: DMAP (0.1 equiv, 0.09 mmol, 0.012 g) was added to a solution of compound **3** (0.9 mmol, 0.56 g) in dry acetonitrile (10 mL) followed by Boc_2O (1.1 equiv) under rapid stirring at rt. The reaction was monitored by ^{1}H NMR until all the reactant had been consumed (18–24 h). *N,N,N′,N′*-tetramethylguadinine (2% in volume) was added, stirring was continued, and the reaction followed by ^{1}H NMR. When all the reactant had been consumed, removal of the solvent at reduced pressure gave a residue that was partitioned between ethyl acetate (50 mL) and $KHSO_4$ (30 mL, 1 M). The organic phase was thoroughly washed with $KHSO_4$ (1 M), $NaHCO_3$ (1 M), and brine (3 × 30 mL, each) and dried with $MgSO_4$. Removal of the solvent afforded an yellow oil (0.39 g, 71 %). Column chromatography (petroleum ether/ethyl ether) gave compound **5** as a beige solid (0,21g, 39 %). ^{1}H NMR (400 MHz, $CDCl_3$,δ):1.31 (9H, s, $C(CH_3)$, 1.53

(3H, d, *J* = 7.2 Hz, CH_3 Npx), 2.92-3.08 (2H, m, β-CH Tyr), 3.62 (1H, q, *J* =7.2 Hz, CH Npx), 3.73 (3H, s, OMe CO_2Me), 3.93 (3H, s, OMe Npx), 4.69-4.74 (1H, m, α-CH Tyr), 5.81 (1H, d, *J* =7.6 Hz, NH Tyr), 6.76 (2H, d, *J* = 8.4 Hz, ArH Tyr), 6.92 (2H, d, *J* = 8.4 Hz, ArH Tyr), 7.10 (1H, s, β-CH ΔPhe), 7.11-7.67 (12H, m, ArH + NH ΔPhe). ^{13}C NMR(100 MHz, $CDCl_3$,δ): 18.12 (CH_3 Npx),28.81 (C(CH_3)$_3$), 36,08 (CH_2 β-CH Tyr), 46.88 (CH Npx), 52.50 (CH_3, OMe CO_2Me), 54.53 (α-CH Tyr), 55.29 (CH_3 OMe Npx), 78.34 (*C*(CH_3)), 105.60 (CH Ar), 119.21 (CH Ar), 123.68 (C ΔPhe), 124.22 (CH Ar), 125.97 (CH Ar), 126.14 (CH Ar), 127.70 (CH Ar), 128.53 (CH Ar), 128.93 (C Ar),129.26 (CH Ar), 129.42 (CH Ar), 129.65 (CH Ar), 130.83 (C Ar),132.51 (C Ar),133.84 (C Ar), 133.81 (C Ar),135.23 (C Ar), 154.30 (C Ar Tyr), 157.79 (C Ar Npx), 165.13 (C=O ΔPhe),169.61 (C=O Tyr) 175.01 (C=O Npx). HRMS (ESI) m/z: $[M+H]^+$ calcd for $C_{37}H_{42}N_2O_6$ 609.3056; found 609.3061.

Synthesis of Npx-L-Tyr-*Z*-ΔPhe-OH, 7: TFA (3 mL) was added to Npx-Tyr(O^{t}Bu)-ΔPhe-OMe, **5** (0.3 mmol, 0.19 g) and the mixture was left at room temperature for 4 hours. The acid was removed under reduced pressure and ethyl ether was added to the residue which was left in the freezer. The solid was filtrated, and the *O*-deprotected dehydrodipeptide Npx-Tyr-ΔPhe-OM,e was isolated as a beige solid (0.14 g, 87%). ^1H NMR (400 MHz, DMSO, δ):1.26 (3H, d, *J* = 7.2 Hz, CH_3 Npx), 2.69-2.99 (2H,m , β-CH_2 Tyr) , 3.60 (3H, s, OMe CO_2Me), 3.81-3.83 (4H, m, OMe Npx + CH Npx), 4.63-4.69 (1H, m, α-CH Tyr), 6.66 (2H, d, *J* = 8.4 Hz, ArH Tyr), 7.07-7.69 (14H, m, ArH Tyr + Npx + β-CH ΔPhe), 8.21 (1H, d, *J*=8.4 Hz, NH Tyr), 9.18 (1H, bs, OH Tyr), 9.79 (1H, s, NH ΔPhe). To a solution of Npx-Tyr-ΔPhe-OMe (0.26 mmol of 0.14 g) in 1,4-dioxane (2mL) NaOH (1M) (3 mL) was added.The reaction was monitered by TLC. The organic solvent was removed under reduced pressure and the reaction mixture was acidified to pH 3 with $KHSO_4$ (1M) the solid formed was filtered affording compound **7** (0.14 g, quantitative yield). ^1H NMR (400 MHz, DMSO-d_6 ,δ):1.24 (3H, d, *J* = 6.8 Hz, CH_3 Npx), 2.67-3.02 (2H, m, β-CH_2 Tyr), 3.75 (1H, q, *J* = 6.8 Hz, CH Npx),3.79 (3H, s, OMe Npx), 4.62-4-87 (1H, m, ,α-CH Tyr), 6.64 (2H, d, *J* = 8.4 Hz, ArH Tyr), 7.07-8.23 (14H, m, ArH Npx + Tyr + ΔPhe), 8.21 (1H, d, *J* = 8.4 Hz, NH Tyr), 9.17 (1H, bs, OH Tyr), 9.6 (1H, bs, NH ΔPhe). ^{13}C NMR(100 MHz, DMSO-d_6 ,δ): 18.91 (CH_3 Npx), 36,59 (β-CH_2 Tyr), 44.66 (CH Npx), 54.19 (α-CH Tyr), 55.12 (OCH_3 Npx), 105.63 (CH), 114.80 (CH Ar), 118.39 (CH Ar), 125.35 (CH Ar), 126.40 (CH Ar), 126.52 (C Ar),126.69 (CH Ar), 127.93 (C Ar), 128.32 (C Ar), 128.34(CH Ar), 128.95 (CH Ar), 129.07 (CH Ar), 129.80 (CH Ar), 130.24 (CH Ar), 131.43 (CH Ar), 133.05 (C Ar), 133.60 (C Ar), 137.14 (C Ar), 155.75 (C Ar), 156.90 (C Ar), 166.19 (C=O ΔPhe), 171.00 (C=O Tyr), 173.32 (C=O Npx). HRMS (ESI) *m/z*: $[M + Na]^+$: calcd for $C_{32}H_{30}N_2NaO_6$ was 561.2072, found: 561.2080.

Synthesis of Npx-L-Asp(O^{t}Bu)-Phe(β-OH)-OMe, 4: Fmoc- Asp(O^{t}Bu)-Phe(β-OH)-OMe, **2** (1.2 mmol, 0.49 g) was dissolved in DMF (2 mL) and piperidine (0.5 mL) was added. The mixture was left stirring for 10 minutes until a white precipitate started to form. Ethyl acetate (50 mL) was added and reaction mixture was washed with water (3 x 50mL). The organic phase was dried and removal of the solvent afforded a mixture of the *N*-deprotected dipeptide with dibenzofulvene as an oil (0.32g). The oil was dissolved in dry DCM (6 mL) and (S)-(+)-naproxen chloride (0.86 mmol , 0.21g) and NEt_3 (2 eq., 1,72 mmol, 0,25 mL) were added. The reaction mixture was left stirring overnight (18 h). Removal of the solvent at reduced pressure gave a residue that was partitioned between ethyl acetate (50 mL) and $KHSO_4$ (30 mL, 1 M). The organic phase was thoroughly washed with $KHSO_4$ (1 M), $NaHCO_3$ (1 M), and brine (3 × 30 mL, each) and dried with $MgSO_4$. Removal of the solvent afforded compound **4** as diastereomeric mixture (0.39 g, 79%). ^1H NMR (400 MHz, DMSO ,δ):1.35 and 1.36 (9H, 2s, C(CH_3), 1.58 and 1.61 (3H, 2d, *J* = 7 Hz, CH_3 Npx), 2.31-2.81 (2H, m, β-CH_2

Asp), 3.57 and 3.65 (3H, 2s, OMe CO_2CH_3), 3.68-3.74 (4H, m, CH Npx) 3.91 and 3.92 (3H, 2s, OCH_3 Npx), 4.63-4.66 [2H, m, α-CH Phe(β-OH) and α-CH Asp), 5.13-524 [1H, m, β-CH_2 Phe(β-OH)], 7.11-7.76 [14 H, m, ArH + NH Asp + NH Phe(β-OH)]

Synthesis of Npx-L-Asp(O^{t}Bu)-Z-ΔPhe-OMe, 6: DMAP (0.1 equiv, 0.07 mmol, 0.009 g)) was added to a solution of compound **4** (0.7 mmol, 0.39 g) in dry acetonitrile (5 mL) followed by Boc_2O (1.0 equiv) under rapid stirring at rt. The reaction was monitored by ^{1}H NMR until all the reactant had been consumed (18–24 h). *N,N,N′,N′*-tetramethylguadinine (2% in volume) was added and the reaction followed by ^{1}H NMR. Removal of the solvent at reduced pressure gave a residue that was partitioned between ethyl acetate (50 mL) and $KHSO_4$ (30 mL, 1 M). The organic phase was thoroughly washed with $KHSO_4$ (1 M), $NaHCO_3$ (1 M), and brine (3 × 30 mL, each) and dried with $MgSO_4$. Removal of the solvent afforded an yellow oil (0.22 g, 56 %). Column chromatography (petroleum ether/ethyl ether) gave compound **5** as a white solid (0,12g, 31 %). ^{1}H NMR (400 MHz, $CDCl_3$,δ):1.36 (9H, s, $C(CH_3)$, 1.61 (3H, d, *J* = 7.2 Hz, CH_3 Npx), 2.55 (1H, dd, *J* = 6.8 Hz e 16.8 Hz, β-CH_2 Asp), 2.89 (1H, dd, *J* = 4.0 Hz e 16.8 Hz, β-CH_2 Asp), 3.73 (3H, s, OMe CO_2Me) 3.76 (1H, q, *J* = 7.2 Hz, CH Npx), 3.92 (3H, s, OMe Npx), 4.88-4.93 (1H, m, α-CH Asp), 6.82 (1H, d, *J* = 8.0 Hz, NH Asp), 7.04 (1H, s, β-CH ΔPhe), 7.11-7.13 (1H, m, ArH), 7.24-7.26 (3H, m, ArH), 7.32-7.37 (4H, m, ArH), 7.61-7.68 (3H, m, ArH), 7.84 (1H, bs, NH ΔPhe). ^{13}C NMR (100 MHz, $CDCl_3$,δ): 18.25 (CH_3-Npx), 27.90 [$(CH_3)_3C$], 36,29 (CH_2 β-CH_2 Asp), 47.00 (CH Npx), 49.62 (CH α-CH Asp), 52.47 (CH_3 CO_2Me), 55.29 (CH_3 OMe Npx), 81.94 [$C(CH_3)$], 119.14 (CH Ar), 123.52 (C ΔPhe), 125.71 (CH Ar), 125.98 (CH Ar), 127.74 (CH Ar), 127.79 (CH Ar), 128.43 (CH Ar), 128.59 (C Ar), 129.01 (C Ar), 129.22 (CH Ar), 129.44 (CH Ar), 129.72 (CH Ar),133.21 (CH Ar),133.26 (C Ar), 133.80 (C Ar),135.69 (C Ar), 157.72 (C Ar Npx), 169.27 (C=O ΔPhe),171.22 (C=O Asp) 174.58 (C=O Npx)**.** HRMS (ESI) m/z: $[M+H]^+$ calcd for $C_{32}H_{37}N_2O_7$ 561,25884; found 561.25953.

Synthesis of Npx-L-Asp-Z-ΔPhe-OMe, 8: TFA (1.5 mL) was added to Npx-Asp(O^{t}Bu)-ΔPhe-OMe, **6** (0.35 mmol, 200 mg) and the mixture was left at room temperature for 4 hours. The solvent was removed under reduced pressure and ethyl ether was added to the residue which was left in the freezer. Compound **8** was obtained as beige solid (144 mg, 82%). ^{1}H NMR (400 MHz, DMSO ,δ):1.41 (3H, d, *J* = 7.2 Hz, CH_3 Npx), 2.59-2.77 (2H, m, β-CH_2 Asp) , 3.59 (3H, s, OMe CO_2Me), 3.81 (1H, q, *J* = 7.2 Hz, CH Npx), 3.83 (3H, s, OMe Npx), 4.73-4.79 (1H, m, ,α-CH Asp), 7.1 (1H, dd, *J* = 2.8 Hz e *J* =9.2 Hz ArH), 7.19 (1H, s, β-CH ΔPhe), 7.21-7.23 (4H, m, ArH,), 7.44 (1H, dd, *J* = 1.6 Hz e 8.8 Hz, ArH Npx), 7.57-7.60 (2H, m, ArH) 7.67-7.71 (3H, m, ArH), 8.39 (1H, d, *J* = 7.6 Hz, NH Asp), 9.59 (1H, s, NH ΔPhe), 12.37 (1H, bs, COOH). ^{13}C NMR(100 MHz, DMSO ,δ): 18.95 (CH_3-Npx), 35,88 (CH_2 β-Asp), 44.70 (CH Npx), 49.55 (α-CH Asp), 52.10 (OCH_3 CO_2Me), 55.14 (OCH_3 Npx), 105.66 (CH Ar), 118.44 (CH Ar), 125.46 (CH Ar), 125.62 (CH Ar), 126.49 (CH Ar), 126.68 (CH Ar), 128.37 (C Ar), 128.46(CH Ar), 129.08 (CH Ar), 129.36 (CH Ar), 130.11 (CH Ar), 132.10 (β-CH ΔPhe), 133.11 (C ΔPhe), 137.08 (C Ar), 156.95 (C Ar), 165.25 (C=O ΔPhe), 170.57 (C=O Asp), 171.51 (CO_2H) 173.63 (C=O Npx). HRMS (ESI) m/z: $[M+Na]^+$ calcd for $C_{28}H_{28}N_2NaO_7$ 527.17887; found 527.17862.

Hydrogel Characterisation

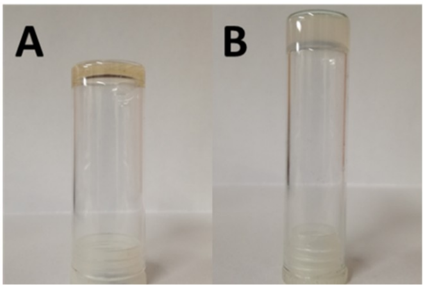

Figure S1. Images of hydrogels: A) **7** (0.4 wt%) and B) **8** (0.4 wt%).

Table SI. Summary of the properties of the SPIONs incorporated into hydrogels **7** and **8**.

**Composition**	**Core size (nm)**	**Hydrodynamic Diameter (HD) (nm)**	**Zeta potential (mV)**	**Saturation Magnetisation (M_s) (emu/g)**
**Fe_3O_4 Magnetite**	**8**	**108**	**-87.2**	**88.9**

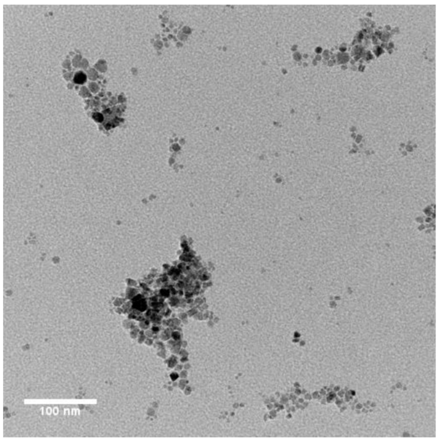

Figure S2. TEM image of SPIONs (scale 100 nm) incorporated into hydrogels **7** and **8**.

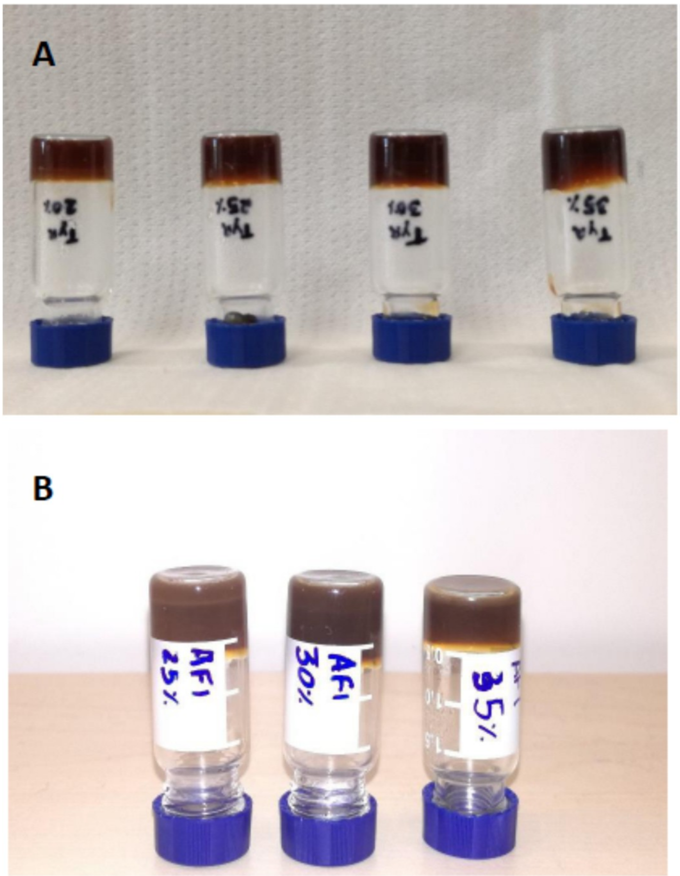

Figure S3. Images of hydrogels **7** and **8** obtained with incorporated SPIONs: A) hydrogel **7** (0.8 wt%) with incorporated SPIONs 20%, 25%, 30% and 35%. B) hydrogel **8** with incorporated SPIONs 25%, 30% and 35%.

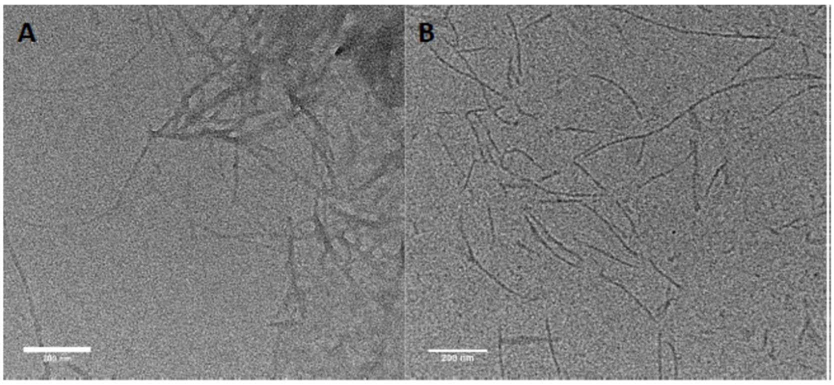

Figure S4. TEM images of: A) hydrogel **7** and B) hydrogel **8** obtained with Uranyless staining (scale bar 200 nm).

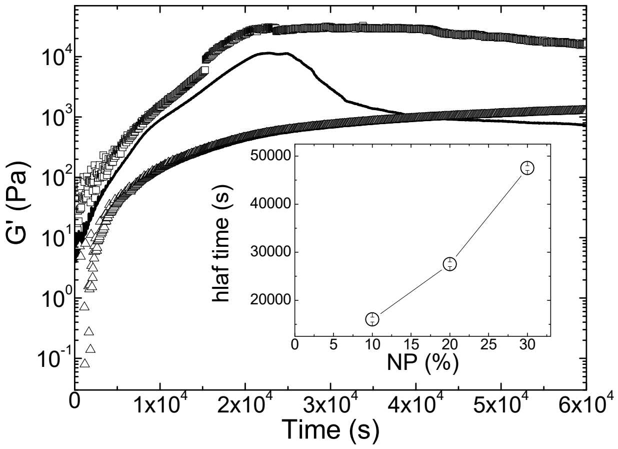

Figure S5. Time evolution of the storage modulus G′ of hydrogel **7** formulated without (squares: data measured with 0.001% strain; line: data measured with 0.01% strain) and with 20 % SPIONs (triangles). Inset: effect of SPIONs content of the half time for gel structural buildup.

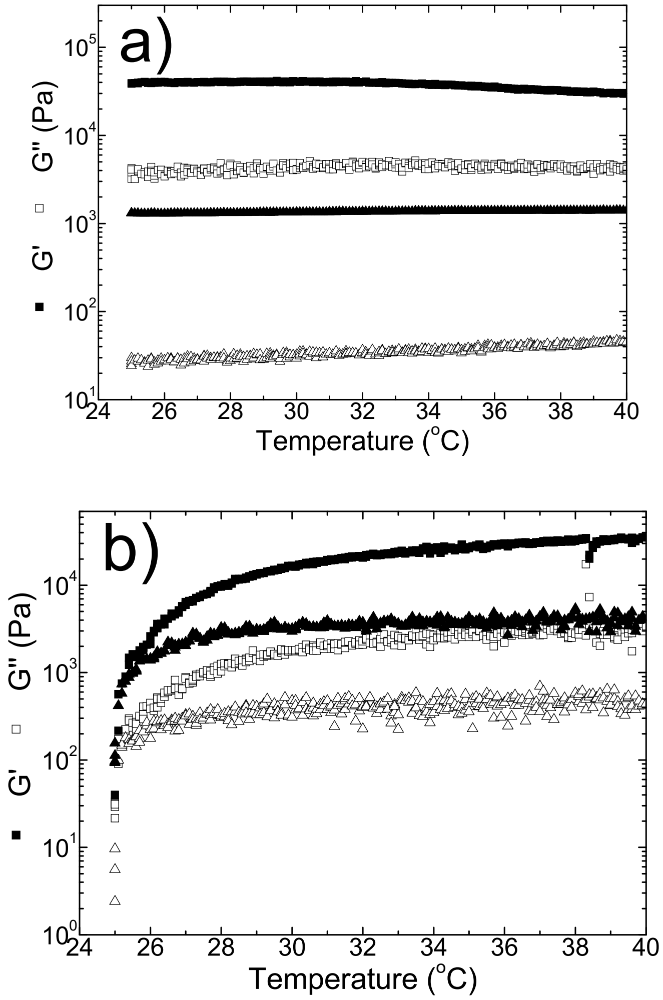

Figure S6. Temperature dependence of the storage (G′, solid symbols) and the loss (G′′, empty symbols) moduli of (a) hydrogels **7** formulated without (squares) and with 20% SPION (triangles), and (b) of hydrogels **8**

formulated without (squares) and with 30% SPION. In (b), gels were submitted to strain sweeps depicted in **Figure S7** prior to the heating ramp, thereby explaining the partial gel recovery at lower temperatures.

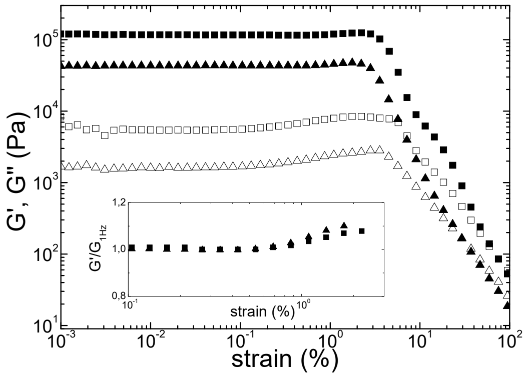

Figure S7. Strain dependence of the storage (G′, solid symbols) and the loss (G′′, empty symbols) moduli of hydrogels **8** formulated without (squares) and with 30% SPIONs (triangles). Inset: strain dependence of the scaled storage modulus G'/G_{1Hz}.

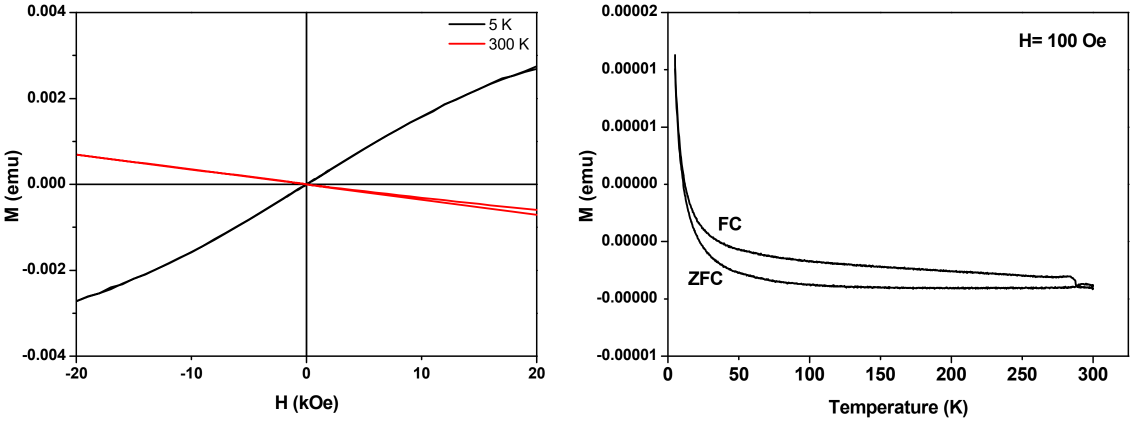

Figure S8. Magnetic characterization of hydrogel **8** (0.8 wt%) without SPION: A) Hysteresis loop at 5 and 300K; B) *ZFC-FC* magnetisation curves (FC, H= 100 Oe).

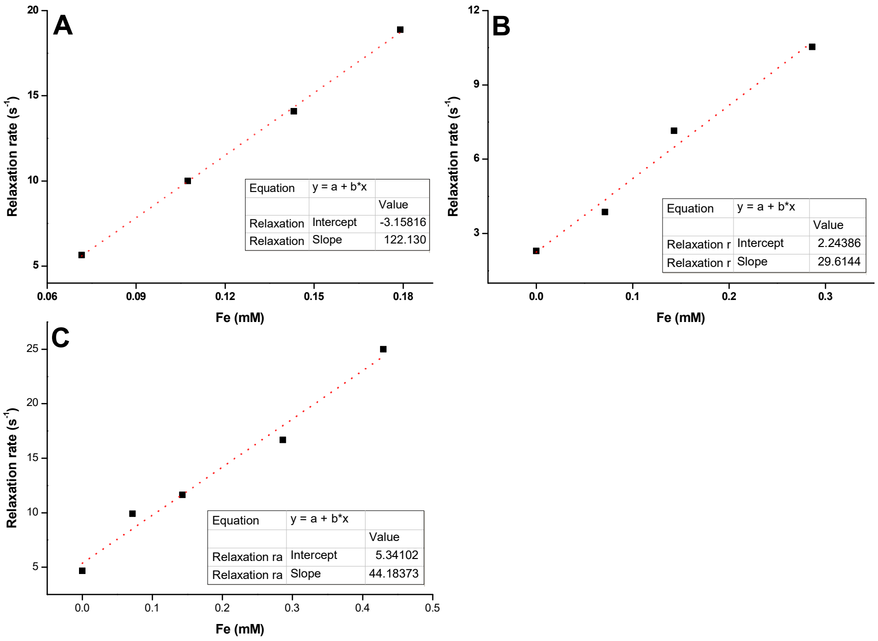

Figure S9. Dependence of the observed paramagnetic transverse relaxation rate (R_2; 3 T, 25 ºC) on the Fe concentration for: A) SPIONs in water; B) SPIONs incorporated into hydrogel **7** (0.8 wt%) and C) SPIONs incorporated into hydrogel **8** (0.8 wt%).

The efficacy of the SPIONs as a paramagnetic water relaxer was evaluated by its transverse relaxivity, r_2 (equation 1).

$$R_{2(obs)} = \frac{1}{T_{2,0}} + r_2 C \quad \text{(eq. 1)}$$

$R_2 = 1/T_2$ (s^{-1}) represents the observed transverse relaxation rate of the water protons; $T_{2,0}$ (s) is the observed diamagnetic transverse relaxation time of water protons (measured before adding the paramagnetic species); r_2 ($mM^{-1}\,s^{-1}$) is the transverse relaxivity and C (mM) is the concentration of the paramagnetic species (Fe).

Table SII. Evaluation of the SAR parameter for SPIONs in water and incorporated into hydrogels **7** and **8**.

SPIONs		Water			Hydrogel **7** 0.8 wt%			Hydrogel **8** 0.8 wt%		
% m/m	m_{Fe} (g)	dT/dt (ºC/s)	ΔT (ºC)	SAR (w/g)	dT/dt (ºC/s)	ΔT (ºC)	SAR (w/g)	dT/dt (ºC/s)	ΔT (ºC)	SAR (w/g)
25	0,0029	0,243	62	**357**	0,109	36	**161**	0,23672	34	**347**
30	0,0034	0,266	68	**325**	0,0822	35	**100**	0,24967	45	**305**
35	0,0056	0,303	73	**227**	0,0875	35	**65**	0,32117	34	**240**

The specific absorption rate (SAR; W/g) was calculated by the initial slope method (equation 2).

$$SAR = \frac{CVs}{m_{Fe}}\frac{dT}{dt} \qquad \text{(eq. 2)}$$

C is the volumetric specific heat capacity of the magnetic nanoparticle solution (J/(cm^3.ºC)); *V*s is the volume of sample (cm^3); m_{Fe} (g) is the mass of iron in the experiment and dT/dt is the initial rate of temperature rise obtained from the *T* vs *t* curves.

Biological studies

Reagents and standards for biological studies - Lipopolysaccharide (LPS) from *Escherichia coli*, sodium pyruvate, sulphanilamide, 3-(4,5-dimethylthiazol-2-yl)-2,5-diphenyltetrazolium bromide (MTT), dimethyl sulfoxide (DMSO), trizma hydrochloride, trypan blue, soybean lipoxygenase (LOX) from *Glycine max* (L.) Merr. (Type V-S; EC 1.13.11.12), propan-2-ol, butan-1-ol, sodium nitroprusside (SNP), bovine serum albumin (BSA), methanol, acetonitrile and formic acid of HPLC purity were from Sigma-Aldrich (St. Louis, USA). Dulbecco's Modified Eagle Medium (DMEM), Modified Eagle Medium (MEM), foetal bovine serum (FBS), 0.25% trypsin-EDTA (1X) and Pen Strep solution (penicillin 5000 units/mL and streptomycin 5000 μg/mL) were purchased from GIBCO, Invitrogen (Grand Island, USA).

Cell culture - RAW 264.7 macrophages were from the American Type Culture Collection (LGC Standards S.L.U., Barcelona, Spain), and MRC-5 cells were from MRC-5 ATCC (Barcelona, Spain). Cells were cultured in DMEM supplemented with 10% FBS and 1% penicillin/streptomycin, and cells were incubated at 37 °C, in a humidified atmosphere of 5% CO_2. Cell passages were kept low for all cell lines, with a maximum of 12 passages.

Biological studies included the *MTT reduction assay*

MTT reduction assay - Cells were seeded in 96-well plates at a density of 25000 and 15000 cells/well for RAW 264.7 and MRC-5 cells, respectively. After 24h, different concentrations of the molecules under study were added and plates were incubated for 24 h. At the end of this period, the metabolic activity of cells was evaluated by their ability to reduce yellow tetrazolium MTT to a purple formazan product. Results are expressed as percentage of the respective control and correspond to the mean ± standard error of the mean (SEM) of, at least, three independent experiments performed in triplicate.

Determination of NO levels - RAW 264.7 cells were seeded in 96-well plates at a density of 35 000 cells/well and incubated for 24 h at 37 °C and 5% CO_2. Afterwards, the medium of each well was removed and cells were pre-treated with different concentrations of 7 or 8 or media only. After 2 h, cells were stimulated with l μg/mL of LPS for 22 h. The quantity of NO in cell culture medium was determined by its conversion to nitrite, using a mixture of 75 μL of culture media with an equal volume of Griess reagent (1% sulphanilamide and 0.1% *N*-(naphth-1-yl)ethylenediamine dihydrochloride in 2% H_3PO_4) in a 96-well plate. The plate was incubated for 10 min, in the dark, at room temperature and the absorbance was measured at 560 nm in a microplate reader (Multiskan ASCENT, Massachusetts, USA)[30]. The results correspond to the mean ± SEM of three independent experiments performed in triplicate and are expressed as percentage of NO in cells exposed to LPS (positive control for NO production).

LOX inhibition assay - Inhibitory effect on 5-lipoxygenase was evaluated according to a previously described method [31]. Briefly, 20 μL of 7/8 solution, 200 μL of phosphate buffer (pH 9) and 20 μL of soybean lipoxygenase (100 U) were added to each well of 96-well plates. After 5 min

pre-incubation at room temperature, 20 µL of linoleic acid (4.18 mM in ethanol) were added and its oxidation to 13-hydroperoxylinoleic acid was followed for 3 min, at 234 nm, in a microplate reader (Multiskan ASCENT, Massachusetts, USA). Three independent experiments were performed in triplicate.

COX-1/COX-2 inhibition assay - The assay was performed using the COX fluorescent inhibitor screening assay kit (Cayman chemical, MI, USA), with some modifications. Briefly, 60 µL of assay buffer (100 mM Tris-HCl, pH 8.0), 5 µL of hemin, 5 µL of enzyme (either COX-1 or COX-2) and 5 µL of EnP(5,8) were added in a black 96-well plate. After 5 min of incubation at room temperature, 5 µL of ADHP and 20 µL of a solution containing arachidonic acid (0.5 mM) and KOH (2.5 mM) were added to each well. After a further 2 min at room temperature, the fluorescence of resorufin was monitored with an excitation wavelength between 530-540 nm and an emission wavelength between 585-595 nm. The results correspond to the mean ± SEM of at least three independent experiments performed in duplicate.

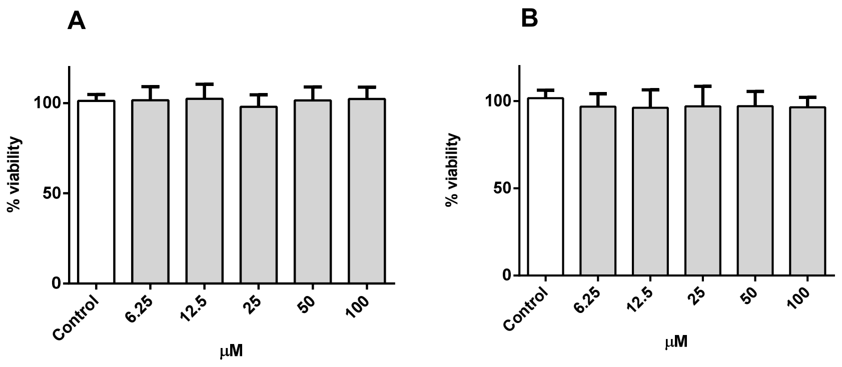

Figure S10. Viability of MRC-5 cells incubated with hydrogelators **7** (A) and **8** (B). Data represent the mean ± standard deviation of the mean of three independent experiments, performed in triplicate.
